# Viruses and neurodegeneration: a growing concern

**DOI:** 10.1186/s12967-024-06025-6

**Published:** 2025-01-12

**Authors:** S. Shouman, N. Hesham, T. Z. Salem

**Affiliations:** 1 Biomedical Sciences Program, UST, Zewail City of Science and Technology, October Gardens, 6th of October City, Giza, 12578 Egypt; 2 Molecular Biology and Virology Laboratory (MBVL), Center for X-Ray Determination of the Structure of Matter (CXDS), Zewail City of Science and Technology, October Gardens, 6th of October City, Giza, 12578 Egypt

**Keywords:** Neurodegenerative diseases (NDDs), Neuropathic infections, Neuroinflammation, Neurotropic viruses, COVID-19

## Abstract

Neurodegenerative diseases (NDDs) cause a progressive loss of neurons. Since NDDs are multifactorial, the precise etiology varies on the basis of the type of disease and patient history. Cohort studies and case studies have demonstrated a potential link between viral infections and the onset or progression of NDDs. Recent findings concerning the mechanisms by which neuropathic infections occur have provided more insights into the importance of such connections. In this review, we aim to elaborate on the occurrence of the neuropathic effects of viruses from epidemiological, clinical, and biological perspectives while highlighting potential treatments and challenges. One of the key players in viral neuropathogenesis is neuroinflammation caused by the immune response to the virus; this can occur due to both neurotropic and nonneurotropic viruses. The COVID-19 pandemic has raised concerns about whether vaccines are essential for preventing viruses or whether vaccines may play a part in exacerbating or accelerating NDDs. By classifying viruses and the common NDDs associated with them and further delving into their cellular pathways, this review provides insights to advance the development of potential treatments and diagnostic methods.

**Graphical Abstract:**

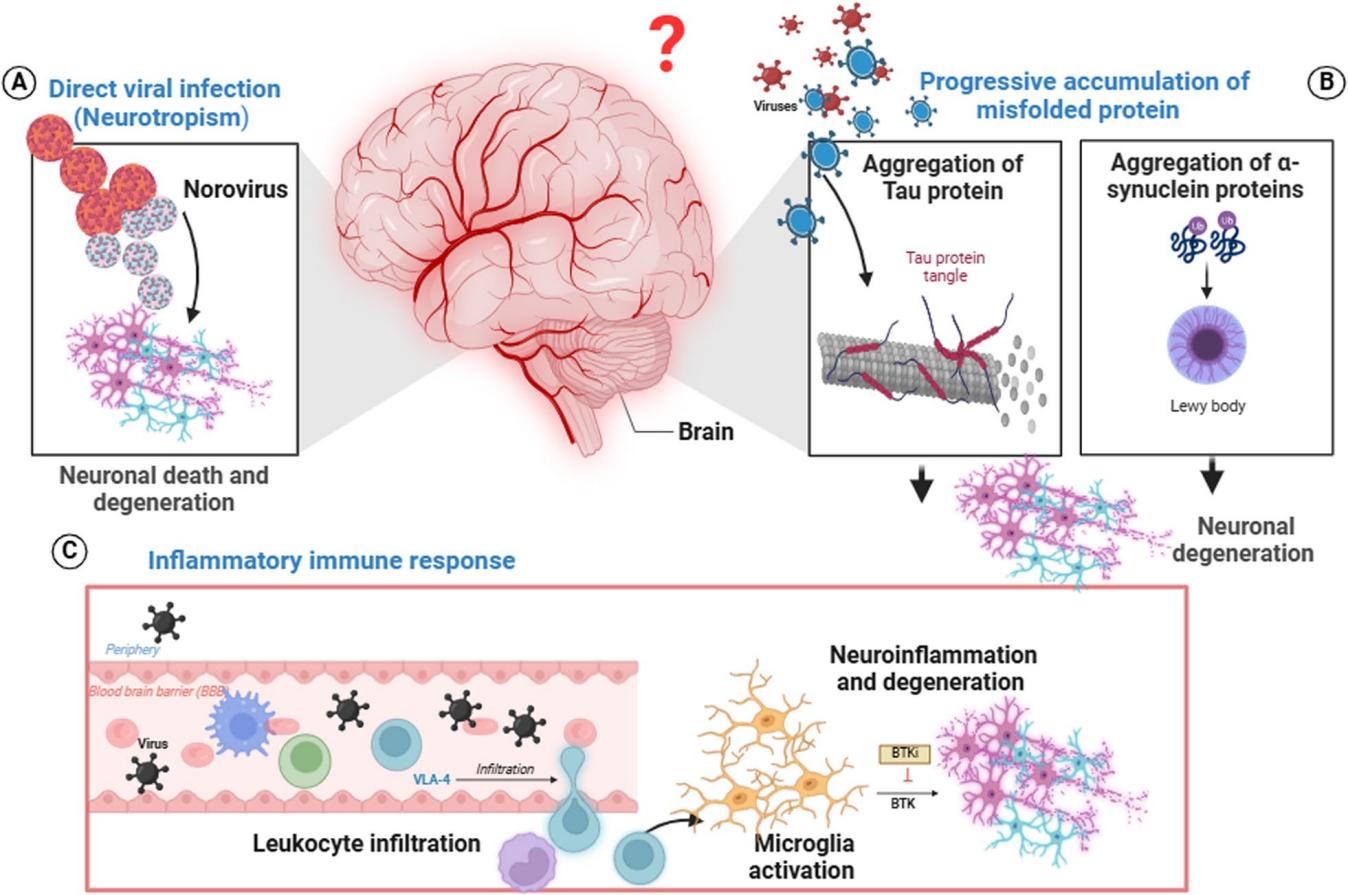

## Introduction

**Fig. 1:**
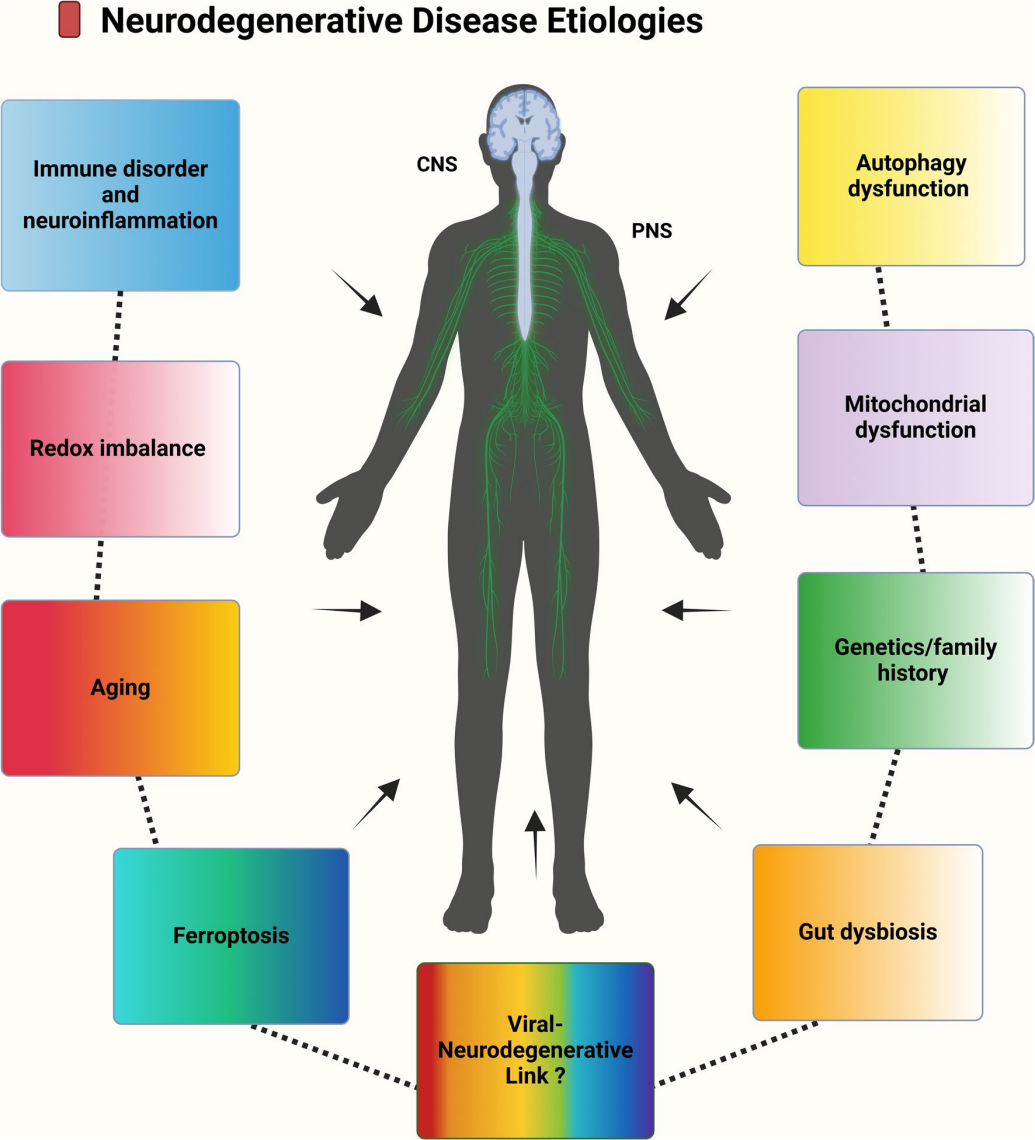
Key Pathological Mechanisms in Neurodegenerative Diseases

Neurodegenerative diseases (NDDs) are the most prevalent disorders of the central nervous system (CNS) in adults. The most common NDDs include Alzheimer’s disease (AD), Parkinson’s disease (PD), Huntington’s disease (HD), and amyotrophic lateral sclerosis (ALS), among others [1]. A long global life span is partially linked to the prevalence of NDDs [2, 3], whereas age remains the primary risk factor [1]. Growing evidence indicates that genetic makeup [4] and environmental factors can also increase the risk for NDDs [5, 6]. However, the exact cause and molecular mechanisms behind the pathogenesis of NDDs still require further research [7]. Notably, the shared pathological mechanisms of neuronal diseases include the accumulation of misfolded proteins or peptides [8], ubiquitin‒proteasome system inactivation, autophagy dysfunction [9, 10], neuroinflammation [11, 12], oxidative stress [13], ferroptosis [14], microbiota dysbiosis [15], and mitochondrial dysfunction [16] (Fig. 1). Recently, several studies revealed that multiple pathogens, such as viruses, are linked to important signaling pathways underlying NDD pathogenesis [17–19]. In many cases, viral infection is easily transmitted, widely distributed, and challenging to control. Viruses can infect neurons directly or indirectly via immune-mediated damage [20, 21]. Epstein–Barr virus (EBV) and human immunodeficiency virus (HIV) are linked to an increased risk of NDDs [22–24]. Recent studies have noted the connection between COVID-19 and neurodegenerative disorders, suggesting a link between viral exposure and neuroinflammation or neurodegeneration [25]. The potential link between NDDs and several neurotropic viral infections, such as herpes simplex viruses (HSVs) [26], poliovirus [27], and rabies [28], is indeed known. Notably, this field is limited by the lack of valid biomarkers for early detection and effective therapeutic approaches [29] [30]. In this review, we cover the common NDDs associated with viral infections, with a focus on their molecular mechanisms and clinical implications, as well as their potential treatments.

## Neurodegenerative diseases and the mechanisms underlying the viral–neurodegenerative link

**Fig. 2:**
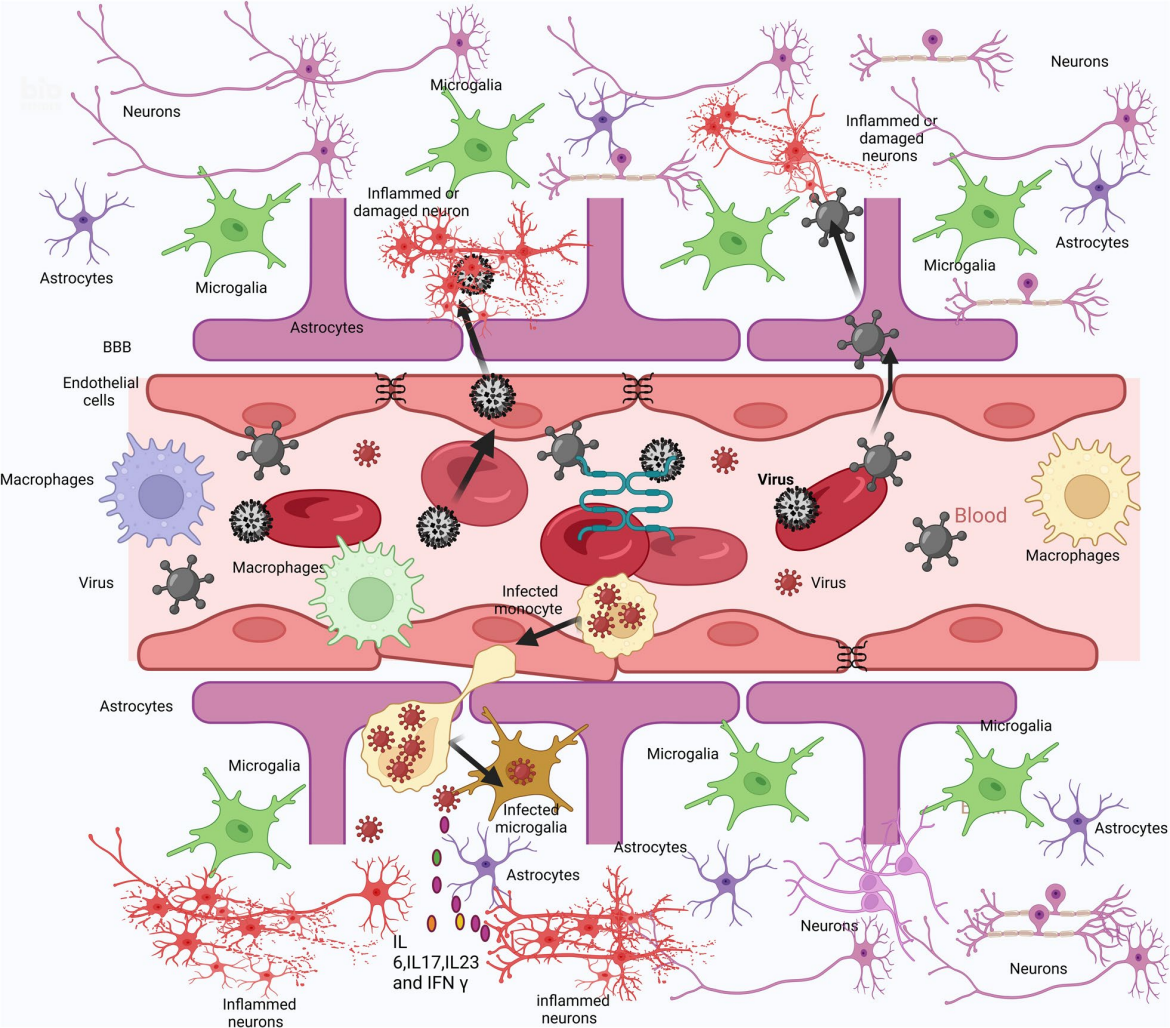
Viral Mechanisms of Blood-Brain Barrier Infiltration

Generally, the blood–brain barrier (BBB) provides immunologically privileged sites to protect the brain. Neurotropic viruses, on the other hand, can circumvent these safeguards and infiltrate the CNS. Even though neurotropic viruses can cross the BBB via different mechanisms (see Fig. 2) [31], it is unclear whether these viruses can directly cause NDDs. Viral infections stimulate sequential immunological responses. However, a protracted immunological reaction results in extensive inflammation, which impairs neuron function. As a result, the cause of neuron loss could be due to the virus or persistent inflammation. The invading virus is recognized by Toll-like receptors. TLR3 recognizes dsRNA [32], and TLR7 and TLR8 recognize single-stranded RNA (ssRNA) [33], whereas TLR9 recognizes nonmethylated CpG islands of DNA viruses [34]. The downstream signaling pathway of TLRs activates NF-κB and interferon regulatory factor (IRF) via IL-1R-associated kinase (IRAK) and the adaptor protein MyD88 [35]. This triggers the production of proinflammatory cytokines and type I IFNs [36]. Although TLRs are expressed mostly on monocyte‒macrophages and dendritic cells (DCs) [37], they are also expressed on astrocytes, microglia, oligodendrocytes, and neurons [38]. Prolonged inflammation can activate M1 proinflammatory microglia together with infiltrating macrophages [39], which exacerbates inflammation, increases neuronal toxicity and may promote protein aggregation. Damaged neurons emit damage-associated molecular patterns (DAMPs) and aberrant proteins, either molecules detected by TLRs, which are subsequently taken to lysosomes for destruction or spread to nearby neurons, culminating in a vicious cycle of neuroinflammation [40]. However, previous research has revealed that viruses may play a direct role in the development of certain NDDs, albeit only in individual casualties. Data from the FinnGen project [41] for more than 300,000 people in Finland as well as the UK Biobank [42] for approximately 500,000 people have been retrieved and analyzed. The study focused on six NDDs: generalized dementia, vascular dementia, Parkinson's disease (PD), Alzheimer’s disease (AD), amyotrophic lateral sclerosis (ALS), and multiple sclerosis. The essential criterion was whether the hospitalized patient had a prior viral illness. Among the viruses, EBV, influenza, herpes zoster, herpes simplex, and enteroviruses were included in the study. In FinnGen, 45 cases presented a 30-fold greater likelihood correlation between viruses and NDDs, whereas 22 cases in the UK Biobank presented a 22-fold greater likelihood correlation. The strongest link was found between viral encephalitis and AD, revealing a correlation but not causation. Another interesting question is whether virus-based vaccines may accelerate or slow the onset of NDDSs. Other evidence linking NDDs with viral infection is discussed below. Neurodegenerative diseases and mechanisms underlying the viral–neurodegenerative links are presented in Table 1.

**Table 1:** Neurodegenerative diseases and mechanisms underlying the viral-neurodegenerative links

Virus	Family	Genome	Disease	Mechanism of pathogenesis	References
Cytomegalovirus (CMV)	Herpesviridae	dsDNA	Alzheimer’s Disease	Chronic inflammation during viral replication	[13]
Herpes simplex virus (HSV)	Herpesviridae	dsDNA	Alzheimers Disease	Associated with higher rate of APOE genotype	[108]
			Parkinson’s Disease	Assumed to be neuroinflammation and immunological factors	[63]
			Amyotrophic lateral sclerosis	Neuroinflammatory	[72]
Human herpesvirus-6 (HHV-6)			Multiple sclerosis	Promotes inflammatory cascade	[85]
Varicella zoster virus			Multiple sclerosis	Viral reactivation could trigger immune responses, in turn causing the demyelination of neurons	[86, 109]
Epstein‒Barr virus (EBV)			Parkinson’s Disease	Antigen mimicry	[66]
Human papillomavirus (HPV)	Papillomaviridae	dsDNA	Dementia	Systemic inflammation and impaired blood flow can lead to cognitive decline	[101, 109]
Human Immunodeficiency virus (HIV)	Retroviridae	ssRNA	Alzheimers Disease	Can infect macrophages in microglia	[51]
			Huntington’s disease	Speed up the appearance of symptoms in HD	[24]
Influenza A	Orthomyxoviri-dae	-veRNA	Alzheimers Disease	Microglial activation due to immune activity against the virus	[52, 109]
			Parkinson’s Disease	Occurrence is associated no mechanism uncovered	[67, 109]
SARS-CoV-2	Coronaviridae	ssRNA	Alzheimers Disease	Hyperinflammation and hypoxia	[53]
			Amyotrophic lateral sclerosis	Neuroinflammation leads to notable decline in cases	[78]
			Huntington's disease	Inflammatory effect caused by the infection can trigger early onset	[93]
			Vascular dementia	Causative agent in the small vessel cerebrovascular complication	[102, 109]
			Friedreich ataxia	Worsens the disease due to the effect on the complications of the disease such as diabetes and cardiomyopathy, sleep apnea and scoliosis	[103]
Hepatitis C virus (HCV)	Flaviviridae	ssRNA	Parkinson’s Disease	Dopaminergic neuronal toxicity	[65]
Epstein‒Barr virus (EBV)	Herpesviridae	dsDNA	Parkinson’s Disease	Antigen mimicry	[66]
			Multiple sclerosis	Promotes inflammatory cascade	[85, 109]
Coxsackievirus B3 (CVB3)	Picornaviridae	ssRNA	Amyotrophic lateral sclerosis	Promoting immune cell infiltration and activation, includes an increase in cytokine and chemokine expression	[77]
Norovirus	Caliciviridae	RNA	Lewy body disease	Ulcerated lesion along with inflammation and fibrosis showed traces of norovirus related to alpha-synuclein	[96]

### Alzheimer’s disease (AD)

**Fig. 3:**
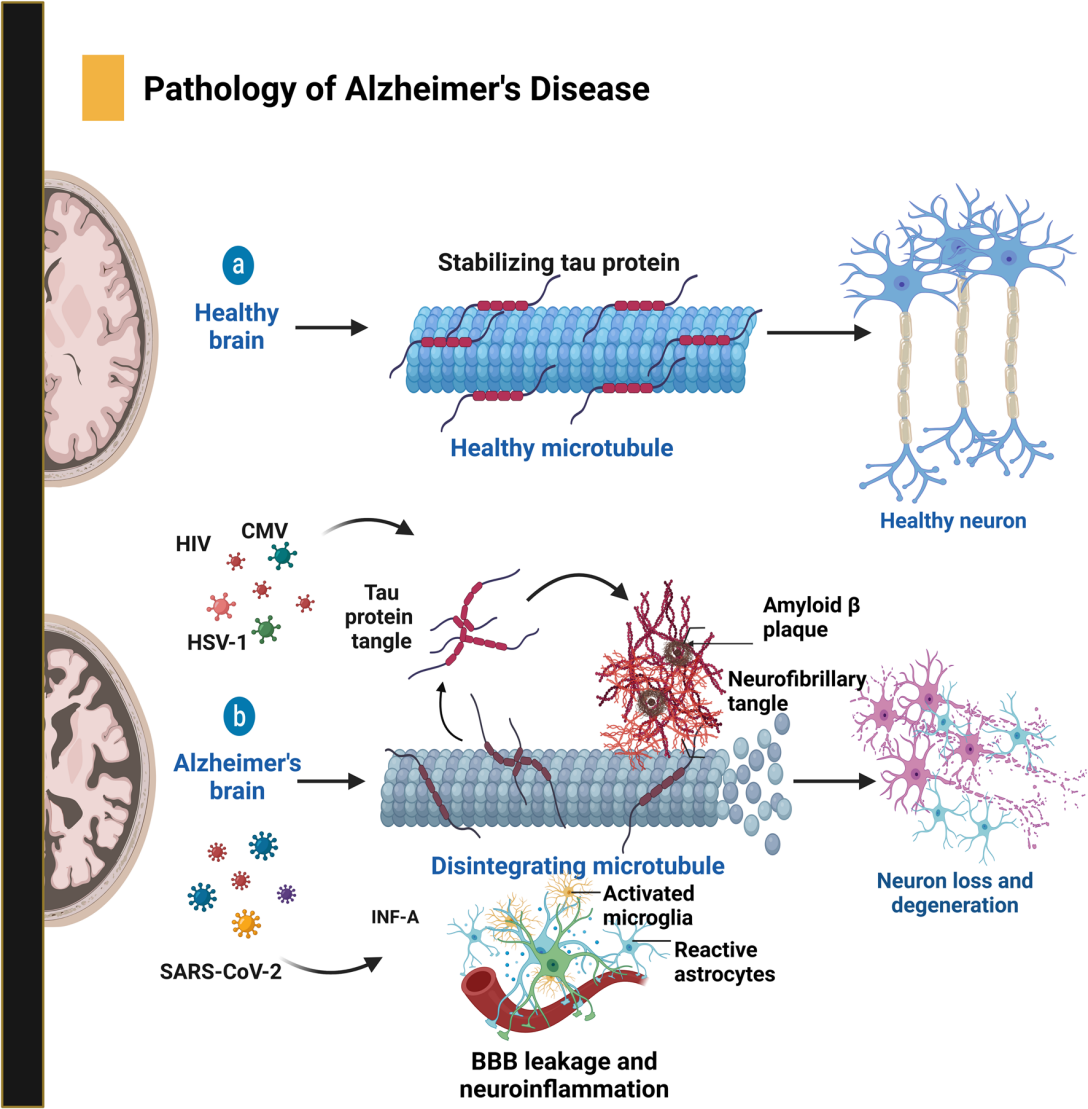
Neuropathology of Alzheimer's Disease and Viral Pre-Infection

The most prevalent neurodegenerative condition associated with age-dependent dementia is AD. However, the underlying cause of AD is enigmatic. Typically, AD is characterized by brain atrophy in the medial temporal lobes (Fig. 3) [43]. The pathophysiologic hallmarks of AD include extracellular amyloid-β (Aβ) plaques and the intracellular aggregation of hyperphosphorylated tau proteins in neurofibrillary tangles (NFTs). However, the activation of glial cells could also play a significant role in the development of AD [44, 45]. Additionally, mitochondrial dysfunction can lead to increased production of reactive oxygen species (ROS) and oxidative stress, which are suggested to contribute to AD development [46]. Various genetic variants associated with AD have been identified. However, most cases are genetically influenced but not considered genetically determined, at least not with Mendelian inheritance [47]. Apolipoprotein E (APOE) is the most common gene linked to late-onset AD. Individuals carrying the APOE*ε4 mutant allele have an increased risk of developing the disease by three- to four-fold, whereas individuals with homozygous alleles have an increased risk of up to 15-fold [48]. A population-based cohort study revealed that the prevalence of AD can be a result of cytomegalovirus (CMV) infection due to the chronic inflammation caused by its replication triggering Amyloid-beta (Aβ) plaque deposition [13]. Alpha-herpes viruses such as herpes simplex viruses 1 and 2 and varicella-zoster virus (VZV) are known to infect the brain, leading to the development of Alzheimer's pathology. According to a population-wide cohort study in Korea, after three herpes viruses were analyzed, HSV-1 and VZV were specifically associated with the development of AD. HSV-1 infection was associated with a relatively high rate of APOE genotype AD, which highlights the influence of HSV-1 and VZV infection at the molecular level [49]. A study using fibroblast and neuronal cells isolated from a mouse and a rat, respectively, tested the effects of murine CMV and HSV-1 infection on the development of AD pathology. The study revealed increased levels of phosphorylated tau for both viruses, which was a result of the activity of the enzyme glycogen synthase kinase 3 beta during HSV-1 infection but not during murine CMV infection. HIV has also been associated with increased Aβ, as the virus can target microglia, and NDDs caused by HIV are classified as HIV-1-associated neurodegenerative disorders (HANDs) [50]. Amyloid precursor protein (APP) has been investigated for its ability to inhibit HIV. The results of the study revealed that the 99-aa C-terminal fragment of APP (C99) can inhibit HIV replication by blocking the Gag element responsible for the structural formation of the virus. However, the virus was able to ubiquitinate C99 and proceed with replication, causing the disease to further develop. This also explains why infection with HIV is capable of increasing toxic amyloid levels [51]. Research concerning influenza A viruses confirmed the antiviral activity of Aβ, indicating that a strong immune response is likely to influence the development of AD pathologies. Further investigations into the effects of long-term chronic neuroinflammation caused by influenza A subtypes revealed that immune activity against the virus was undoubtedly behind microglial activation due to inflammation [52]. The levels of AD biomarkers, such as p-tau181, t-tau, GFAP, NfL and UCHL1, in COVID-19 patients were positively correlated with disease severity. Some COVID-19 symptoms, such as hyperinflammation and hypoxia, are also linked to the incidence of AD [53]. Another study of COVID-19 patient brain lysates identified leaky RyR2 as a target to treat AD-like symptoms. Inflammatory and oxidative stress mainly results in tau hyperphosphorylation. Patients have repressed expression of the calbindin protein in the brain, particularly in the cortex and cerebellum, increasing susceptibility to Ca2^+^ dysregulation due to leaky channels [54]. Growing concerns have been raised for COVID-19-vaccinated recipients because the extended amino acid sequence of the spike protein, which is customary for prion-like proteins, may cause neurodegeneration [55]. It has been reported that AD is exacerbated after COVID-19 infection due to endocytosis of the spike protein and its interaction with amyloid-β and hyperphosphorylated tau [56].

### Parkinson’s disease (PD)

Parkinson's disease (PD) is the second most common neurological disease worldwide after Alzheimer’s disease. The development of Parkinson's disease may be linked to environmental factors, genetic variables, and toxins, which could serve as initiating factors for the brain [57]. The clinical hallmarks of PD include the accumulation of alpha-synuclein (α-syn) in Lewy bodies and the loss of dopaminergic neurons in the substantia nigra (SN) of the brain [58]. The immune system and related inflammatory processes are increasingly being implicated in the onset of PD. In particular, microglia and T cells are activated in response to misfolded α-synuclein aggregates in dopaminergic neurons, resulting in their death [59]. In addition to the aforementioned factors, oxidative stress, mitochondrial failure, ferroptosis, and gut dysbiosis have all been implicated in PD pathological pathways [57]. It has been hypothesized that the LRRK2 gene, which is linked to inflammatory bowel illness, could modulate inflammatory pathways linked to neurodegeneration [60]. Owing to the complex and variable nature of PD pathobiology among individuals, finding effective treatments remains challenging. Currently, available evidence suggests that there is a possible role for viruses in the development of PD; however, this topic remains debatable [61]. A population-based case‒control study revealed an inverse relationship between the occurrence of PD and infection with an alpha herpesvirus or CMV, a beta herpesvirus, suggesting that herpesvirus infection might reduce the likelihood of developing PD. Alpha herpesviruses remain dormant in the peripheral nervous system, which might account for this neuroprotective property when accessing the CNS. The exact underlying protective mechanism is still unknown, especially to explain the inverse relationship with CMV infections, as they do not remain dormant in the CNS [62]. On the other hand, a retrospective cohort study revealed the opposite findings regarding herpes zoster virus, another alpha herpesvirus. Although the pathogenic basis was unable to be determined because of the nature of the study, it is assumed to be related to neuroinflammation and immunological factors [63]. Hepatitis C virus (HCV) was found to infect the CNS after the HCV RNA levels in the brain were tested [64]. A study examining the epidemiology of HCV occurrence and PD, as well as investigating molecular pathology, confirmed that HCV can trigger the development of PD [65]. In this study, HCV caused dopaminergic neuronal toxicity in an HCV-infected midbrain culture, characterized by an increase in three chemokines: sICAM (responsible for TNFα-based inflammation), LIX (aiding demyelination), and RANTES (playing a role in neuronal apoptosis). Additionally, there was an unusual downregulation of TIMP-1, a neuroinflammatory marker crucial for cell survival, illustrating the ability of the virus to inhibit neuroprotective properties. The results revealed a similar effect to that of the 1-methyl-4-phenylpyridinium (MPP^+^),, a chemical used to induce Parkinson's disease. One study reported that the virus does not take the metabolic syndrome path through the cytokines released because of insulin resistance; rather, it enters through the blood‒brain barrier [65]. PD patients have a significant number of α-syn autoantibodies in their serum. Epstein–Barr virus (EBV) infections are linked to PD, as findings show that infected people can produce antibodies that cross-react with α-syn because antigen mimicry autoantibodies are generated against EBV, confirming its role in the subsequent aggregation of α-syn and the progression of PD [66]. The results from a ten-year case‒control study in Denmark revealed a positive correlation between influenza infections and PD diagnosis. Other types of infections, such as pneumonia and respiratory tract infections, did not yield significant results, indicating that their conclusions are more specific [67].

### Amyotrophic lateral sclerosis (ALS)

Amyotrophic lateral sclerosis (ALS), also known as Lou Gehrig's disease, is a progressive neurodegenerative disorder that affects motor neurons in the brain and spinal cord. ALS leads to the loss of voluntary muscle control, eventually causing paralysis and, in most cases, death [68]. ALS can be classified into two types: familial and sporadic. Sporadic ALS is the most common form, accounting for approximately 95% of cases, and occurs randomly without a clear family history. Familial ALS is inherited and occurs in certain families, resulting in the remaining cases [69]. Despite the number of studies carried out on ALS, its pathogenesis is still unclear [70]. Recently, TAR-DNA binding protein (TDP-43) was identified as a key component of ubiquitinated aggregates in ALS [71].

An animal model was used to detect neuroinflammatory changes caused by latent HSV infection. A depleted amount of C9orf72 was found, as ALS is characterized by a loss-of-function mutation in C9orf72; this leads to speculation of the role of HSV, a known neurotropic virus, in the development of ALS [72]. Another study highlighted the role of human endogenous retroviruses (HERVs) and tested the abundance of HERV viral elements in patients and revealed the impact of the virus on disease pathogenesis. In this study, HERV-K induced neurotoxicity through env protein expression. TDP-43, which forms cytoplasmic aggregates characteristic of ALS, can bind to the virus’s LTR region, leading to its transactivation [73]. PEG10, a domesticated retrotransposon gag-pol protein, specifically accumulates in spinal cord tissue and is linked to transcriptional changes that play a role in the pathophysiology of ALS. The role of this domestic retrotransposon adds to the evidence of the effect of viral elements on the development of neurodegenerative disorders [74]. However, further research on the biology of PEG10 and its abundance is needed to provide more context. Neurotropism caused by enteroviruses has been proven during the study of several neurological diseases [75, 76]. According to an in vivo study in a mouse, coxsackievirus B3 (CVB3), an enterovirus, was able to trigger the onset and exacerbate the symptoms of ALS through promoting immune responses, including immune cell infiltration and activation, and increasing cytokine and chemokine expression [77]. A notable decline in two ALS patients was observed after SARS-CoV-2 infection; this decline was speculated to be a result of neuroinflammation. The study revealed the importance of vaccination as a preventative measure to protect ALS patients from severe decline [78]. New-onset ALS in a patient after receiving the J&J COVID-19 vaccine was also reported. This finding was explained by the potential triggering of the neuroinflammatory response; the patient included in the study had a family history of ALS, indicating a probable genetic predisposition that might have been triggered by the neuroinflammatory effects of the vaccine. This finding contradicts the findings of previous studies calling for the vaccination of ALS patients, as vaccination could increase neuroinflammation [79].

### Multiple sclerosis (MS)

Multiple sclerosis (MS) is a chronic neurodegenerative disease that affects the central nervous system (CNS), including the brain and spinal cord, in young adults. The etiology of MS is not fully understood, but it is generally recognized as an autoimmune disease in which immune cells attack the myelin sheath surrounding nerve fibers. This immune-mediated damage interferes with the normal transmission of electrical impulses along the nerves [80]. However, it is believed that a combination of environmental and genetic factors contributes to susceptibility to MS. Recent research has identified potential interactions between specific factors in the initiation of MS. MS risk modulators, such as genetic variants in interleukin-2 receptor-α (IL2RA*T), interleukin-7 receptor-α (IL7RA*C), MGAT1 (IVAVT-T) and CTLA-4 (Thr17Ala), as well as environmental factors affecting vitamin D3 levels, converge to alter the branching of *N*-glycosylation [81]. Consequently, a reduction in branching leads to T-cell hyperactivity and promotes spontaneous inflammatory demyelination of nerves in an animal model [82]. However, the exact mechanisms underlying the initiation and progression of MS remain elusive. EBV and human herpesvirus-6 (HHV-6) are thought to be the most relevant viruses with respect to multiple sclerosis (MS) onset and progression [83]. The connection between MS and viral infections is complicated, and evidence suggests that MS can be triggered by a viral infection through observing the success of interferon therapies [84] and MS patient screenings. Both EBV and HHV-6 belong to Herpesviridae, and multiple studies have proven their association with MS through the inflammatory MS cascade [85]. Another study explains that, like other alphaherpesviruses, the varicella-zoster virus (VZV) establishes latency in neuronal cells. This leads to an association between the reactivation of the virus and MS relapse. This finding can be further explained by the observation that treatment of MS with immunosuppressants can trigger the reactivation of VZV. However, this reactivation contributes to the progression of MS. It was proposed that this happens due to the inflammation and autoimmune response causing more demyelination [86]. It remains uncertain if VZV virus can be definitively labeled as a cause of MS [86]. A cohort study in Brazil revealed their roles in promoting relapsing–remitting MS (RRMS) and primary progressive MS (PPMS) by detecting viral gene expression in patients. High HHV-6 accompanied by lower EBV infection was reported; both viruses were found at the highest frequency among females with RRMS [85]. Some of the proposed mechanisms linking herpesviruses with MS progression include the ability of EBV-infected T cells to cross-react with self-antigens, leading to CNS damage, and bystander activation in the CNS due to the presence of viral antigens. Another mechanism linking EBV to MS involves the production of autoantibodies by autoreactive B cells. A high-frequency peptide of the EBNA-1 EBV antigen shares homology with the N-terminus of αB-crystallin, which is expressed in MS lesions [87].

### Huntington's disease

Huntington's disease (HD) is caused by an increase in the number of CAG repeats in the huntingtin gene (HTT). The greater the number of repeats, the earlier the onset and more severe the symptoms tend to be. In particular, neurons in the striatum of the brain are the most susceptible to death. HTT is a large intracellular protein of 350 kDa that is ubiquitously expressed and primarily localized in the cytoplasm [88]. Because HTT contains both nuclear export and nuclear localization signals, the protein actively shuttles between the nucleus and the cytoplasm [89]. HTT is crucial in CNS development, including the formation of neural tubes (NTs) and neuroblast migration. HTT also contributes to axonal transport, synapse function, and cell survival [90]. Knockout of HTT in mice leads to death shortly after nervous system formation before birth [91]. The mutated Htt easily aggregates, thereby promoting endoplasmic reticulum (ER) stress, which in turn leads to neuronal injury and apoptosis [92]. There is currently no cure for HD, and various treatments and interventions can help manage symptoms. During the COVID-19 pandemic, a case study of two patients revealed that they developed early symptoms of Huntington’s disease, which implies that the inflammatory effect caused by the infection can trigger early onset of the disease [93]. A retrospective analysis of the effect of HIV infection on the onset of Huntington’s symptoms revealed that infection seems to accelerate the appearance of symptoms in HD patients, resulting in an earlier age of onset [24]. It appears that certain viruses can influence the pathogenesis of the disease; however, this was previously observed through the detection of the onset of symptoms.

### Lewy body disease

Lewy body disease (LBD) is characterized by the presence of aberrant protein aggregates, called Lewy bodies, that develop in neuronal cells in the brain. Its symptoms vary between cognitive decline, visual hallucinations, and motor symptoms [94]. Lewy body disease pathology is thought to be initiated in the gut and nose by a pathogen that enters through the nasal cavity [95]. An autopsy of a patient who suffered from Lewy body disease revealed norovirus infection in the intestinal mucosa as well as alpha synuclein aggregates. An ulcerated lesion along with inflammation and fibrosis showed traces of norovirus related to alpha-synuclein, and the study concluded that the virus may have exacerbated disease development in addition to being the cause [96].

### Dementia

Vascular dementia (VaD) is the second leading cause of cognitive dementia in elderly individuals after Alzheimer's disease. Both the clinical phenotypes and pathogenetic mechanisms of these diseases are heterogeneous. Generally, a progressive deterioration in cognitive abilities, including reasoning, planning, and remembering, is a clinical hallmark. In addition, secondary focal neurons are damaged because of a diminished cerebral blood supply to the brain. Several risk factors contribute to the onset of VaD. These include age, high blood pressure, smoking, diabetes, high cholesterol, and a history of strokes or cardiac diseases [97, 98]. The treatment of VaD is limited due to its unexplained causes. Currently, there are no approved standard treatments for VaD [99]. Infectious diseases are known to increase the risk of dementia, with the highest risk being vascular dementia [100]. To link HPV infection with the incidence of vascular dementia, it was found that oxidative stress due to soluble IL-2 in HPV patients can cause ischemic stroke, which increases the risk of VaD. A previous study revealed that the inflammatory effects of HPV can impair blood flow to the brain, also contributing to the risk of dementia [101]. An observational study of COVID-19 patients studying the conversion of mild cognitive impairment into dementia revealed rapid progression in acute infections. These results lead to the identification of COVID-19 as a main causative agent of small vessel cerebrovascular complications and call for the targeting of neurovascular uncoupling as a target to prevent the progression of dementia from mild-cognitive impairment in COVID-19 patients [102].

### Other NDDs less likely to be associated with viral infections

Some NDDs lack evidence of a correlation between the onset of disease and viral infection. This may be due to the limited data available for rare neurodegenerative diseases or the predominantly genetic etiology of the disease. The neurodegenerative diseases that fall under this category are spinal muscular atrophy (SMA), Friedreich ataxia (FRDA), Batten disease, and creutzfeldt–Jakob disease, which are discussed below. A study examining the possible effects of COVID-19 on FRDA revealed that the disease seems to worsen when coupled with SARS-CoV-2 infection due to its effect on complications such as diabetes and cardiomyopathy, sleep apnea and scoliosis [103]. As a disease caused purely by genetic factors, there are limited data regarding the influence of viral infections on the disease. Creutzfeldt–Jackob disease (CJD) is the most common human prion disorder and comprises rare diseases known as transmissible spongiform encephalopathies (TSEs). These disorders are characterized by the abnormal accumulation of misfolded proteins, called prions, in the brain. Creutzfeldt‒Jakob disease affects both humans and animals. The clinical manifestations of CJD are rapid mental deterioration, involuntary muscle spasm and dementia. There is no cure or treatment for CJD, and most people die within a year [104]. While sporadic CJD occurs spontaneously and is not thought to be transmissible between individuals, iatrogenic CJD can result from medical procedures involving contaminated materials. Variant CJD is caused by prion proteins. These proteins are responsible for bovine spongiform encephalopathy (BSE) in cattle, which can cause cow disease [105]. However, the findings of an early case study revealed that inflammation in brain tissue occurred in the presence of HSV infection in a Creutzfeldt‒Jakob disease patient, and it was speculated that the disease might have stimulated the virus in the latent phase in the CNS [106]. Owing to limitations in sporadic HIV cases, a study reported no correlation between the virus and the disease [107].

## Potential therapeutic strategies

**Fig. 4:**
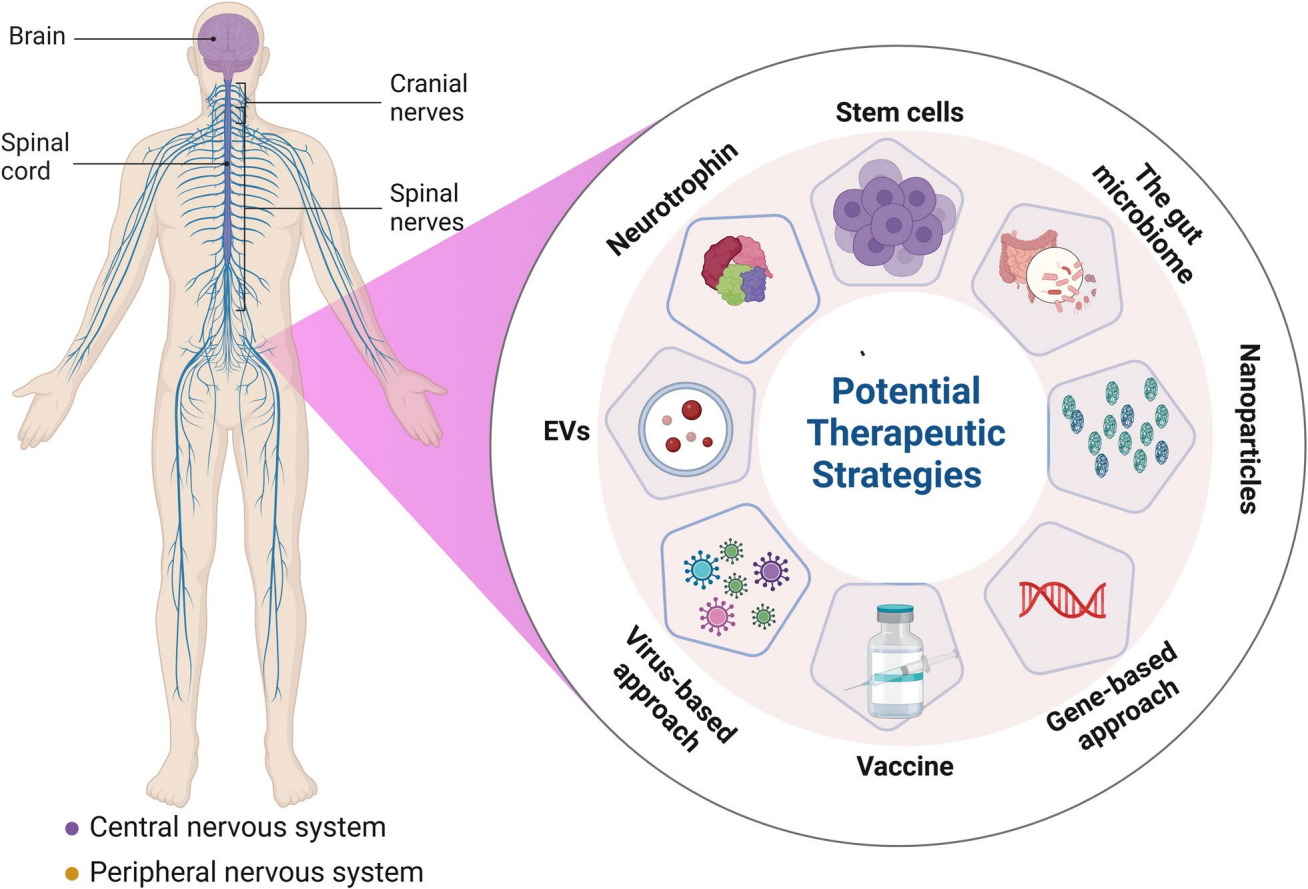
Therapeutic Strategies for Neurodegenerative Diseases

Viral-associated neurodegenerative diseases present notable challenges, and therapeutic approaches are often tailored to the virus involved. The initial strategy involves controlling infectious viruses to target noninfectious conditions such as neurodegenerative disorders [108]. The development of vaccines against neurotropic viruses can help reduce the incidence of associated NDDs. Immunotherapeutic strategies targeting specific aberrant proteins, such as the Aβ and tau proteins, in Alzheimer's disease have been clinically approved [109]. Aducanumab is an anti-Aβ human IgG1 monoclonal antibody-based immunotherapy that targets Aβ aggregates. This monoclonal antibody is designed to bind specifically to both soluble and insoluble aggregated forms of Aβ. It was the first drug licensed by the US Food and Drug Administration (FDA) for the treatment of Alzheimer's disease, under the Accelerated Approval pathway. It is noteworthy that, the approval was controversial, as the FDA’s advisory committee was not informed that the drug would be considered for accelerated approval during their review process. However, the required confirmatory trial to verify clinical benefits is expected to be completed by 2030. It remains debatable whether targeting Aβ directly translates to cognitive improvement in Alzheimer's disease patients. Nonetheless, Aducanumab's approval has influenced the landscape of Alzheimer's research, despite the lack of clear clinical benefits. [69, 110]. In addition, immunotherapies for hyperphosphorylated tau, hyperactivated microglia, and α-syn aggregates are also under development [111]. Below are some general strategies and considerations for managing virus-associated NDDs (Fig. 4).

### Immunotherapy in NDDs

To date, a line of scientific effort has been aimed at designing highly effective vaccines capable of initiating specific immunity against abnormal protein aggregates. The most well-known defaulted proteins associated with NDDs are the Aβ, tau, Htt and α-syn proteins. In 2000, the first active immunotherapy for AD treatment was the clinical trial (AN-1792). AN-1792 was designed to work by supplying Aβ peptides into the bloodstream, where they serve as immunogens to provoke an anti-Aβ T-cell-mediated immune reaction [112]. The formed plaques (Aβ40, Aβ42 and Aβ43) were successfully cleared from AD patients. This vaccine, however, could neither slow nor improve the rate of cognitive decline. Approximately 19 patients had severe meningoencephalitis. This was explained by the infiltration of antibodies and/or T cells into the brain. Because of these negative side effects, the AN-1792 trials have been halted [113, 114]. To avoid this undesired response, chimeric Aβ vaccination with norovirus particles presenting the Aβ immunogen has been proposed. This technique greatly increases immunogenicity and antibody production without activating Aβ42-specific T cells [115]. Another approach is the creation of active immunization for AD with the help of peptide fragments along with the QS-21 adjuvant with immunogenic particles. It also has the advantage of not eliciting Aβ42-specific T cells, unlike when long-chain peptides are used [116]. Another strategy for treating AD is to target phosphorylated tau protein. In Japan, the first clinical trial was conducted using BIIB092, a humanized IgG4 monoclonal antibody, to neutralize the N-terminus of tau [117]. There were no deaths or major adverse effects, and BIIB092 at doses of up to 210 mg suppressed unbound N-terminal tau for three months [117]. However, the US authorities later denied approval of this drug because it did not induce any changes in the patient’s cognitive behavior during a two-year trial [118]. Its usage is currently limited to testing in animal models. JNLP3 transgenic mice carry a transgene encoding human tau with four microtubule-binding repeat domains, and three anti-phosphorylated tau antibodies (RZ3, CP13, and PG5) were used for AD therapy. Only the CP13 antibody, which binds to phosphorylated serine residue 202 of the tau protein, significantly decreases insoluble or soluble tau species in the cortical and hindbrain regions of transgenic mice [119]. In PD, targeting only α-syn by immunization therapy might not be sufficient to improve the disease course. Several phase I and phase 2 clinical trials have been performed to test the efficacy of anti-α-syn aggregation N- and C-terminal epitopes. PD patients were randomly assigned to receive either low- or high-dose short peptide PD01A- or PD030A-formulated vaccines as the first human clinical phase. Both types of vaccination result in a strong B-cell response to α-syn-targeting epitopes but prevent autoreactive T-cell mobilization [120]. PRX002, a humanized IgG1 monoclonal antibody, binds to the C-terminal region of α-syn and reduces free serum α-syn after a single infusion in phase II clinical trials with good tolerability [121]. However, it failed to fulfill the primary objective of the study to slow the progression of PD and thus terminated. Cinpanemab (BIIB054) is a monoclonal antibody that targets the N-terminus of α-syn. Cinpanemab demonstrated favorable safety, tolerability, and pharmacokinetic profiles in a phase I investigation of healthy controls and Parkinson's disease patients [122]. However, the phase II trial was stopped because cinpanemab did not increase motor rating scale scores [123]. NAbs-α-syn, naturally occurring anti-α-syn autoantibodies, were purified and extracted from intravenous immune globulin (IVIG) from healthy individuals and utilized to immunize an A53T transgenic PD mouse model to develop a novel passive immunization strategy. NAbs-α-syn dramatically decreased the levels of soluble α-syn, α-syn oligomers, and intracellular phosphorylated α-syn deposits. NAbs-α-syn reduced memory and motor impairments, as well as neuroinflammation, in a PD mouse model [124]. In Huntingtin disease, current research on active and passive immunizations against mutant Huntingtin (m-Htt) proteins is inconclusive and unclear. A trial of a potential plasmid vaccine delivered to HDR6/2 mice revealed no influence on the quantity of m-Htt aggregates [125]. Another study investigated peptide, protein, and DNA plasmid vaccines for m-Htt and reported that vaccination with a mixture of three nonoverlapping HTT exon 1 peptides resulted in substantial antibody generation. However, the study did not evaluate the vaccine's efficiency in decreasing m-Htt aggregates [126]. Vaccination against viral infections such as EBV would be beneficial in preventing virus-related diseases such as MS. Clinical trials for two EBV vaccines began in 2022. The first NIH trial of a gp350-ferritin nanoparticle in adults with a Matrix-M adjuvant (clinical trial identifier, NCT04645147) is currently underway. The second trial (clinical trial identifier, NCT05164094) involves testing an mRNA-based vaccine that encodes gp350, gH, gL, and gp42, which are key proteins found in the EBV envelope. These proteins play a critical role in targeting different parts of the EBV life cycle, including viral attachment, fusion, and entry into host cells [127]. The soluble EBV gp350 vaccine could not prevent virus infection, but it did lower the risk of infectious mononucleosis (IM) [128]. MS can occur due to an adverse immune response to the EBV virus; therefore, a vaccine that improves immune control or provides prevention of IM, even if it may not fully stop infection, could reduce the risk of developing MS. Notably, the development of immunization therapy to improve neurodegenerative disorders confronts several hurdles, including the need for precise targeting, potential adverse effects, and disease complexity. Furthermore, the field is evolving, and continuous study may result in breakthroughs and adjustments to current therapeutic techniques.

### Stem cell-based approaches

Stem cell-based therapy has only recently been found to be an effective approach for treating NDDs. Nonetheless, the progress that has been made regarding the use of stem cells to encase or treat NDDs arising from viral infection is relatively in its developmental phase. There are different classifications of stem cells according to their origin and differentiation potency. These include but are not limited to embryonic stem cells (ESCs), progenitor cells, mesenchymal stem cells (MSCs) and induced pluripotent stem cells (iPSCs) [129]. Different approaches are being pursued for stem cell-based therapy for NDDs. The primary approach involves the use of stem cells, neural progenitors/neural stem cells and engineered adult cells either to replace or support damaged neuron circuits through various mechanisms, including immune system inhibition and the secretion of neurotrophic factors. In the case of viral infection, stem cells, particularly iPSCs, can be genetically modified to increase their resistance to viral infections. This may involve introducing genes that confer resistance to specific viruses. IPSCs can be efficiently engineered with the latest methods of genome editing and can be transplanted into autologous patients. The first clinical trials for the treatment of several NDDs via engineered iPSCs were recently approved [130]. However, the primary challenge of cell-based therapy is immunological compatibility. Xu and coworkers knocked out human leukocyte antigen (HLA) genes in engineered iPSCs, making them immune compatible with more than 90% of the global population [131]. The homing capacity of transplanted MSCs for treating traumatic brain injury (TBI) has recently improved. These engineered MSCs that overexpress CXCR4 are highly attracted by the strong chemoattractant stromal cell-derived factor-1 (SDF-1), which is upregulated in astrocytes and endothelial cells in the affected lesion areas. The SDF-1/CXCR4 axis triggers the homing of MSCs to injury sites in the brain and other organs [132]. In addition, neural progenitor cells (NPCs), which reside in the subventricular zone (SVZ) of the adult brain, can migrate to the injury site in the CNS. The response of NPCs is driven by SDF1, a chemokine that regulates NPC migration, and its receptor, CXCR4. This signal is a homing signal that ultimately leads to neuronal integration and CNS repair [133] [134]. The challenge with viral infections is how transplanted MSCs influence T-cell responses and clear EBV and CMV infections. Although MSCs have shown immunosuppressive properties [135], transplanting MSCs into immunocompromised patients may impair immune responses to viruses; hence, the susceptibility to infection is increased. Thus, the MSCs exhibited a double-edged sword. When an intracellular pathogen invades a cell, cytotoxic CD8^+^ T cells secrete IFNγ, which stimulates bone marrow MSCs to produce IL-6. In turn, IL-6 reduces Runx1 and C/EBPα expression in hematopoietic progenitor cells, leading to increased myeloid lineage differentiation and infection clearance. Zhang et al. evaluated the safety and immunological responsesof human umbilical cord-derived MSC (UC-MSC) treatment in HIV-1-infected individual classified as “immunodiscordant responders” or “immunological nonresponders (INRs)” [136]. UC-MSC transfusions favorably increased the number of circulating naïve and central memory CD4 + T cells and restored HIV-1-specific IFN-γ and IL-2 production in INRs. These increases in immune repair were also related to decreases in systemic immune triggers and inflammation in vivo. UB-MSC transfusions are effective and can successfully expand host immune reconstitution in INRs, indicating that such treatments may be used as another immunotherapeutic approach to reverse immune disorders in HIV-1-infected INRs [136]. Recently, MSC transplantation has been verified in clinical studies to treat SARS-CoV-2 infection [137–139]. In phase II trials, patients with severe COVID-19 were transplanted with UB-MSCs. Compared with the placebo group, the treated group presented a smaller lung lesion volume. MSCs were also correlated with a greater percentage of normal CT images and a lower incidence of symptoms in the one-year follow-up of SARS-CoV-2-infected patients. MSC therapy did not influence the development or maintenance of neutralizing antibodies in COVID-19 patients after one year of follow-up [139]. These encouraging outcomes are attributed to MSC immunomodulation, improved repair and/or renewal, and reduced viral reproduction via cell‒cell interactions and paracrine activity. Although recent studies have shown promising cell-based therapies for a wide range of NDD experimental models, there are still substantial challenges that must be solved before any possible applications in human clinical trials. The principles that maintain both the healing potency and the long-term organizational integration of any transplanted cell type remain elusive. The cell-homing capacity, reconstruction of three-dimensional (3D) brain architecture, sufficient numbers of viable cells, and route of administration are all issues that must be addressed before cell-based therapies can be used in clinical trials and treatments for various NDDs.

### Extracellular vesicles (EVs)

Extracellular vesicles (EVs) are nanoparticles released after endosome fusion with the plasma membrane (exosomes) or from plasma membrane-derived vesicles (microvesicles) via the multivesicular body (MVB) pathway. EVs are responsible for cell-to-cell communication [140]. In addition, EVs have been described as vectors in pathological and physiological conditions and could hence be used for vaccine or drug delivery [141]. The term “exosome” was originally used to describe vesicles ranging in size from 40 to 1,000 nm that are released by many cell types [142]; however, the subcellular origin of these vesicles remains undecided. This term was later applied to 40–100-nm vesicles released during reticulocyte development [143]. Exosomes are composed of a lipid bilayer membrane and carry substances such as proteins, DNA fragments, microRNAs (miRNAs), and long noncoding RNAs (lncRNAs) to shuttle between cells [144, 145]. In a study by Zitvogel et al. exosomes were found to be released by B lymphocytes and dendritic cells through a similar route [146]. Platelets, cytotoxic T cells (CTLs), B cells, mast cells, neurons, Schwann cells, oligodendrocytes and intestinal epithelial cells are among the other cell types that have been shown to release exosomes [147–149]. In recent years, interest in investigating the therapeutic and diagnostic capacity of EVs, especially for NDD treatment, has increased. EVs are small vesicles with low immunogenic properties. However, the BBB is one of the hurdles for NDD treatment. Many therapeutic agents have difficulty entering the CNS because of the existence of the blood‒brain barrier (BBB). EVs have been demonstrated to cross the BBB from the brain to the bloodstream and vice versa. Therefore, EVs could provide a potential means of delivering therapeutic cargo to the CNS and treating related diseases [150]. Upon viral infection, EVs can be utilized by the virus in favor of their infectivity and pathogenicity [151, 152]. Gould et al. used the term "Trojan Horse" to describe HIV and exosomes [152]. The author hypothesized that HIV has evolved to utilize the exosome system to infect cells by packaging its viral genome within exosomes [152]. A human cerebral microvascular endothelial (hCMEC) 3D cell line was used to determine whether HIV-1 increases the release of EVs. Compared with that in controls, amyloid-β (Aβ) cargo was elevated in infected hCMEC organoids [153]. Notably, brain endothelial cell-derived EVs successfully transported Aβ to astrocytes and pericytes, crossing the BBB into the brain. On the basis of these findings, the authors suggested that HIV-1 promotes the release of brain endothelium EVs loaded with Aβ, which may increase Aβ exposure to neurovascular cells and lead to amyloid accumulation [154]. Other viruses, such as HCV, HTLV, EBV and Rift Valley fever virus (RVFV), can transfer viral proteins to target cells through EVs [155–157]. The goal is that viral antigens in exosomes increase persistence by hiding viral genomes, evading the immune system, and even improving viral infectivity. EV-enclosed miRNAs are among the most common targets that viruses employ to activate astrocytes and microglia, causing neuronal injury. HIV can activate astrocytes to release EVs containing miR-9, resulting in microglial migration, inflammation, and neuronal death [158]. Japanese encephalitis virus (JEV) infection of microglia also increases the expression of let-7a and let-7b in their secreted EVs. Consequently, let-7a/b modulates microglia-mediated inflammation via interaction with the NOTCH-TLR7 pathway to activate caspases and neuronal apoptosis [159]. To control viral infection, nanoengineered EV systems are widely used in drug delivery. The proposed miRNA-401, which targets HSV-1 ICP4 mRNA, is packaged into modified EVs and transported to virus-susceptible cells, establishing an antiviral environment for at least three consecutive days [160]. A specific antibody for EBV can also be packaged into engineered EVs to selectively target these infected cells [161]. Zou et al. engineered exosomes expressing a single-chain variable fragment (scFv) of high-affinity HIV-1 Env-specific monoclonal antibody-10E8 and loaded them with curcumin or apoptosis-inducing miR-143 to target HIV-1-infected cells or tissues. As a result, HIV-1 virus-infected cells were effectively eliminated [162]. Therefore, changes in the composition of EVs occurring after infection by viruses could be potential diagnostic biomarkers for viral infection [162]. The use of EVs for combating NDDs is an attractive approach, but it is still in its infancy. Many obstacles, such as cargo specificity, standard development, and safety concerns, must be addressed before broad clinical application. Nevertheless, the use of EVs to treat NDDs is an exciting avenue for future research and advancement.

### Neurotrophins

Neurotrophins (NTs), also known as neurotrophic factors, are proteins essential to neural plasticity and development, and they have been a subject of interest in the treatment of neurological disorders [163]. The signaling cascades of NTs are highly important to the CNS in terms of maintaining CNS homeostasis. Research has shown that the occurrence of viral infections in the CNS contributes to the disruption of regulatory mechanisms that allow NT signaling [164]. These findings indicate the importance of the use of neurotrophins in the treatment of neurodegenerative diseases, particularly those that are influenced by viral infections.

### Gene-based approaches

The development of molecular techniques has provided a deeper understanding of neurodegenerative disorders at the genetic level. Many identified mutations have been found to cause the onset of neurodegeneration, leading to the development of platforms to combat them [165]. Gene-based treatments for neurodegenerative disease aim for the goal of permanent correction of these conditions. One successful example of an FDA-approved method is SMA gene therapy, which replaces the defective gene causing the disease [166]. Some of the viruses mentioned previously influence the occurrence of neurodegeneration through neuroinflammation; therefore, gene therapies targeting neuroinflammation can be applied to patients who have a neurodegenerative disease linked to a prevalent viral infection. Having developed a gene therapy that targets astrocytes, a study used brain-specific IL-2 as a neuroprotective agent. The therapy resulted in a slow increase in regulatory T cells following high expression of IL-2. As a result, the experimental mice were protected from multiple sclerosis and other brain injuries, demonstrating that IL-2 gene delivery has protective effects against neuroinflammation [167]. Other research on Alzheimer's disease gene therapy utilized knockdown therapy targeting CD33, a transmembrane sialic acid-binding receptor found on microglia of patients with AD. The upregulation of CD33 is associated with amyloid plaque formation via neuroinflammation. The use of microRNAs to knock out CD33 resulted in a decrease in amyloid-β in mouse models as well as a decrease in proinflammatory cytokines and chemokines [168]. A study investigating a metabolic disorder characterized by MAGE Family Member L2 (MAGEL2) deficiency revealed that this deficiency is related to neuroinflammation in the brain. Researchers have used an adenoviral vector to deliver brain-derived neurotrophic factor (BDNF), which reverses neuroinflammation [168, 169]. Gene delivery of attenuated erythropoietin decreased ocular pressure by lowering neuroinflammation and oxidative stress in patients with glaucoma, a degenerative disease that may cause blindness [170].

### Virus-based approaches

Neurodegeneration involves the loss of neurons in the CNS, and viral vectors have been highlighted over the years as effective tools to deliver therapeutic genes because of their small particle sizes [171]. Compared with retroviruses, adenovirus vectors are favored when a viral vector is chosen because of its small size, simplicity, and ability to infect neurons, which are nondiving, in contrast to retroviruses, which infect only dividing cells. A phase II trial targeting AD used an intracerebral injection of an adeno-associated viralvector (AAV2) to deliver neurotrophic growth factors. The treatment was tolerated and safe; however, it was not effective. The study claims that the reasons for this were a lack of accurate gene targeting and a small sample size [172]. A trial in which AAV2 gene therapy was used to treat PD delivered the human aromatic L-amino acid decarboxylase (AADC) enzyme through magnetic resonance imaging (MRI)-guided adenoviral vector delivery. The results of this trial showed success in tolerating the treatment and showed the potential to improve the patient’s motor function [173].

### Nano-based approaches

CNS disorders are generally highly important for researchers around the world because of the complexity of the disease. Some of the issues encountered when testing pharmacological treatments are toxicity, lack of specificity, and especially crossing the blood‒brain barrier. Recently, nanoengineering has been employed to transform drugs, allowing them to cross the blood‒brain barrier. Nanotechnology can also allow the targeting of particular cells and the delivery of genes, potentially improving neuronal regeneration [174]. The use of nano-based treatments to alleviate neuroinflammation could address neurodegeneration caused by viruses. In a study aiming to employ nanotechnology in the treatment of neuroinflammation, actively targeting endothelial cells by linking anti- Intercellular Adhesion Molecule 1 (ICAM-1) or anti-Vascular cell adhesion protein 1 (VCAM-1) antibodies to liposomes facilitated the effective accumulation of conjugated liposomes in the brain [175]. In efforts to develop a nano-based antiviral drug that targets neuro-AIDs, a bioavailable antiretroviral drug was administered through the nasal route, allowing the drug to cross the blood–brain barrier [176].

### Microbubbles and ultrasound approaches

The use of ultrasound and microbubbles in the treatment of neurological diseases has shown great success; ultrasound can be used to open the blood–brain barrier at a precise location in a reversible manner without causing damage to cells. To find a treatment for AD, a study used retroviral microbubbles to deliver brain-derived nerve growth factor (BDNF) alongside ultrasound to disrupt the blood‒brain barrier. This increased transfection efficiency and BDNF expression overall have therapeutic value for the treatment of AD in vivo in rat models [177]. Macrophages residing in the brain are inflammatory cells and can be referred to as activated microglia/macrophages. Through phagocytosis, they can clear damage; however, if this damage persists for too long, it can lead to neuronal damage. To target this inflammation, ultrasound-targeted microbubble destruction using phosphatidylserine (PS)-modified microbubbles (PS-MBs) was tested. PS is intended to inhibit inflammation caused by macrophages by mimicking the signals produced by apoptotic cells. Microbubbles increase the uptake of PS by activated microglia/macrophages [178]. This technique can be particularly useful in treating NDDs that are concurrent with or caused by a viral infection. The treatment of neurological pathologies caused by HIV faces delivery challenges, rendering it “incurable”. One study opted to use specific receptor binding, ultrasound, microbubbles, and magnetic fields as methods to overcome the blood‒brain barrier [179]. Microbubbles were found to help treat this disease by delivering nerve growth factors. In the case of neurodegenerative disorders, they can also potentially deliver antiviral treatment to the brain in the case of a concurrent infection.

### The gut–brain axis: new therapeutic approach

The gut microbiota directly or indirectly interacts with the enteric nervous system (ENS) [180], enteroendocrine system [181], and immune system [182], facilitating signal transmission via the spinal nerves, vagus nerve, and circulatory system to the CNS. Microbes can also modulate neuroimmunoendocrine function through the secretion of metabolites such as short-chain fatty acids, trimethylamine-N-oxide, amino acids, and tryptophan [183]. Gut dysbiosis may contribute to the pathogenesis of various NDDs [184]. However, the precise mechanisms by which altered gut microbiota influence the CNS are unknown. NDDs associated with gut dysbiosis have led researchers to investigate the clinical uses of microbiome-based treatments such as prebiotics, postbiotics, probiotics, and fecal microbiota transplantation (FMT) [185]. Several studies have shown that the human gut microbiota is a major predictor of the plasma metabolome and is potentially more influential than genetics is [186]. Interestingly, there is a case report of an 82-year-old male patient with Alzheimer's disease who had *Clostridium difficile* infection. The patient received a single FMT infusion with stool from his 85-year-old wife, who was mentally normal. Even two months after FMT, the patient's AD symptoms improved rapidly [187]. Although the majority of changes in bacteria have been identified, certain studies have revealed changes in the gut virome that are related to diseases [188, 189]. The human virome is dominated by bacteriophages, with the most common types being Caudovirales and Microviridae [189]. Prophages can be found in more than 80% of bacterial genomes. Consequently, bacteriophages may play an essential role in influencing bacterial diversity and function and thereby human health [190]. Mayneris-Perxachs et al. reported that increased amounts of Caudovirales bacteriophages are associated with enhanced memory and executive functions of the brain in their gut microbiome study [191]. Additionally, the gut microbiome can be influenced by nutrition. On the other hand, phage therapy has great potential for use in clinical applications. Despite many concerns and difficulties, therapeutic phages must be selected and manufactured on demand as a customized treatment, rendering them impractical as standard off-the-shelf medical products [192]. Manufacturing good manufacturing practice (GMP)-certified treatments is both expensive and time-consuming, which further complicates the launch of phage-based clinical trials [193].

## Challenges, controversies and unanswered questions

Establishing a direct cause‒and‒effect relationship between viral infections and NDDs is challenging. While some studies suggest an association, the debate continues over whether viruses may directly cause neurodegeneration or overactivate host immune reactions [194]. The immune response to viral infections in the CNS is complex. Nonetheless, the immune system is necessary for controlling viral infections; however, an excessive or inadequately regulated immune response might contribute to the development of neurodegenerative diseases [195]. Research is still ongoing to determine how to balance the protective effects of immunity and its potential damaging effects on NDDs. Additionally, establishing whether there is any relationship between viral infections and the onset of NDDs has not yet been completely elucidated. According to some studies, viral infections can act as triggers for neurodegenerative diseases. On the other hand, certain studies suggest that viral infections may exploit existing vulnerabilities within the nervous system [196–198]. The most significant fact is the genetic diversity and evolving nature of viruses. This raises the question of whether there is a common viral pathway leading to neurodegeneration or if different viruses contribute through distinct mechanisms [199]. Finally, studying the associations between viruses and NDDs frequently involves significant ethical considerations, particularly when involving human participants [200]. Specifically, clinical trials have evaluated the safety and efficacy of immunization strategies in human subjects. Importantly, while immunization therapy holds promise, there is currently no widely accepted immunization-based treatment for NDDs [201]. Machine learning algorithms, such as EVEscape, which detects changes in viral genomes that could implement viral immune evasion tactics, have proven invaluable for pandemic prediction [202]. The development of an AI tool that can predict the ability of a virus to trigger the development of an NDD could lead to better treatment plans.

This is a dynamic field, and ongoing research is addressing challenges and refining strategies to harness the potential of the immune system against these complex conditions. Furthermore, methodological variations among research studies and differences in sample sizes, disease models, onset stages, and ethical constraints can all contribute to contradictory results and interpretations.

## Data Availability

All the data presented in this review are available and presented in the text.

## Abbreviations

NDDs: Neurodegenerative diseases
CNS: Central nervous system
EBV: Epstein–Barr virus
HIV: Human immunodeficiency virus
GMP: Good Manufacturing Practice
AADC: L-amino acid decarboxylase
VZV: Varicella-zoster virus
HSVs: Herpes simplex viruses
NTs: Neurotrophins
AD: Alzheimer’s disease
MAPs: Microtubule-associated proteins
Aβ: Amyloid-beta
BDNF: Brain-derived nerve growth factor
BBB: Blood‒brain barrier
IVIG: Intravenous immune globulin
MSCs: Mesenchymal stem cells
EVs: Extracellular vesicles
MVB: Multivesicular body
PD: Parkinson’s disease
MS: Multiple sclerosis
FMT: Fecal microbiota transplantation
CTLs: Cytotoxic T cells
ssRNA: Single-stranded RNA
TLR: Toll-like receptor
HAND: HIV-1-associated neurodegenerative disorders
CMV: Cytomegalovirus
HLA: Human leukocyte antigens
iPSCs: Induced pluripotent stem cells
ESCs: Embryonic stem cells
UB-MSCs: Umbilical cord-derived mesenchymal stem cells
HCV: Hepatitis C virus
CVB3: Coxsackievirus B3
FDA: Food and Drug Administration
ALS: Amyotrophic lateral sclerosis
HD: Huntington's disease
HTT: Huntingtin gene
SDF-1: Stromal cell-derived factor-1
LBD: Lewy body disease
SMA: Spinal muscular atrophy
FRDA: Friedreich ataxia
VaD: Vascular dementia
3D: Three-dimensional
α-syn: Alpha-synuclein
SN: Substantia Nigra
Aβ: Amyloid-β
BSE: Bovine spongiform encephalopathy
DAMP: Damage-associated molecular patterns
RRMS: Relapsing–remitting MS
PPMS: Primary progressive MS
HERVs: Human endogenous retroviruses
ER: Endoplasmic reticulum
